# The association between healthy lifestyle score and risk of metabolic syndrome in Iranian adults: a cross-sectional study

**DOI:** 10.1186/s12902-023-01270-0

**Published:** 2023-01-17

**Authors:** Mahdi Vajdi, Arash Karimi, Mahdieh Abbasalizad Farhangi, Abnoos Mokhtari Ardekani

**Affiliations:** 1 Department of Community Nutrition, Faculty of Nutrition, Tabriz University of Medical Sciences, Tabriz, Iran; 2 Nutrition Research Center, Tabriz University of Medical Sciences, Tabriz, Iran; 3 Tabriz Health Services Management Research Center, Tabriz University of Medical Sciences, Tabriz, Iran; 4 Endocrinology and Metabolism Research Center, Institute of Basic and Clinical Physiology Science & Physiology Research Center, Kerman University of Medical Sciences, Kerman, Iran

**Keywords:** Metabolic syndrome, Obesity, Lifestyle factors, Healthy lifestyle score

## Abstract

**Background:**

Lifestyle risk factors, such as obesity, physical inactivity, smoking, and having an unhealthy diet are significantly associated to the risk of developing metabolic diseases such as type 2 diabetes, insulin resistance, and metabolic syndrome (MetS). Consequently, it is important to examine multiple lifestyle risk factors instead of single ones. Moreover, the combined effects of these factors on risk of MetS are not fully investigated. This study investigated the relationship between HLS and MetS and its components among apparently healthy adults living in Tabriz-Iran.

**Methods:**

This cross-sectional study includes 347 apparently healthy adults aged 20–50. To assess healthy lifestyle, four habits were used to examine HLS: physical activity (PA), smoking, healthy eating index-2015 (HEI-2015), and body mass index (BMI). Smoking status, PA, and dietary intakes (147-item) of study participants were evaluated using valid questionnaires. The lifestyle score ranged from zero (non-healthy) to four (most healthy) points. Binary logistic regression with adjusted models was used to examine the association of HLS and MetS, and its components. *P* < 0.05 was considered statistically significant.

**Results:**

In the current study, the average age and BMI of study participants were 40.78 ± 9.23 years old and 29.23 ± 5.97 kg/m^2^, respectively. Participants in the highest quartile of HLS had significantly lower waist circumference (WC), BMI, weight, and had less fasting blood sugar (FBS), and triglyceride (TG) compared to the lowest quartile of HLS. Individuals in the second quartile of HLS had 24% greater odds for having MetS compared with those in the first quartile (adjusted OR: 1.24; 95% CI: 1.05–2.66). A significant reduction in hyperglycemia **(**adjusted OR: 0.85, 95% CI: 0.55–0.96) and high TG (adjusted OR: 0.83; 95% CI: 0.55–0.93) risk was also observed in the highest category of HLS compared to the lowest category. Moreover, after taking potential confounders into account, low score of HLS was associated with increased odds of MetS in women (adjusted OR: 1.32; 95% CI: 1.03–2.91).

**Conclusion:**

The results showed that the lowest adherence to HLS was associated with higher odds MetS in women, after adjusting for all covariates. Moreover, adherence to a healthy lifestyle was related to lower odds of hyperglycemia and high TG in Iranian adults. To confirm these results, prospective studies are required.

## Introduction

Metabolic syndrome (MetS) (also known as syndrome X or insulin resistance syndrome) is a multicomponent disease characterized by central obesity, lipid and insulin dysregulation, and hypertension [1, 2]. The prevalence estimates vary because of the differences in definitions of this syndrome. Over the last decade, the prevalence of MetS has augmented in developed countries to about 25% of the adult population [3] and the spread of MetS is has been estimated to be 23.8% among Iranian adults [4]. It has been recognized that MetS increases the occurrence of cardiovascular disease (CVD) and coronary heart disease (CHD), and also increases the risk of cognitive decline [5].

The pathogenesis of MetS is multifactorial, with the interplay of nutritional, environmental, and genetic factors and recent studies have proposed that dysregulation of lipid metabolism, insulin resistance and inflammation play important roles in the pathogenesis of MetS [6–9]. Lifestyle risk factors such as obesity, smoking, inactivity, and unhealthy eating patterns, along with population aging, are considered major risk factors for MetS [10–12]. Moreover, previous studies show that lifestyle risk factors are related to each other [13–16]. In previous studies, lifestyle risk modification has been proposed as an effective approach in the prevention of chronic diseases namely obesity, MetS, diabetes, and CVDs; therefore, it has been suggested that the levels of each of lifestyle risk factors can be associated with the risk of developing MetS [13–17]. Consequently, it is important to examine multiple lifestyle risk factors instead of single ones [7]. Due to the fact that multiple lifestyle risk factors are more detrimental to health compared with single one, the identification of risk groups might assist the development of specific health promotion plans for different population groups [13, 18, 19].

Recently, some studies have evaluated the combined effect of lifestyle factors, i.e., regular physical activity, not smoking, healthy dietary patterns, and avoiding obesity as part of the variables called healthy lifestyle score (HLS) with the hazard of several chronic diseases [20–25]. Studies have also shown that high adherence to healthy eating index (HEI), which is one of the ingredients of HLS, decreases the peril of developing CVDs, diabetes, and MetS [26, 27]. Based on the results of the studies, adherence to HLS is related to improving overall health in old age [28], reducing the risk of coronary artery disease [29], MetS-related diseases, and decreasing mortality in type 2 diabetics [30, 31]. A longitudinal study carried out in 2017 in the SUN cohort of youth to middle-aged adults found that the HLS was associated with a significant reduction in CVD [32, 33]. Moreover, according to a study by Garralda-Del-Villar et al. [7] healthy lifestyle decreased the progression of MetS. A recent cohort study in Iran showed that greater adherence to HLS was associated with a reduced risk of six-year incidence of MetS [34]. Notably, they determined the HLS by three procedures (HLS-AHEI-2010, HLS-modified French Programme National Nutrition Santé-Guideline Score, HLS-healthy diet pattern score), whereas previous studies have evaluated the HLS of participants in a single way [29, 31]. However, they have only examined the association between HLS and risk of MetS and did not examine MetS components among men and women separately. In another study conducted in Iran, Farhadnejad et al. [23] reported higher score of HLS, characterized by normal body weight, no smoking, vigorous physical activity, and healthy diet, is associated with decreased risk of type 2 diabetes incidence. As far as we know, there has been no cross-sectional study across Iranian adults has been conducted to examine the association between HLS and the risk of MetS and its components.

As mentioned above, there is a direct relationship between lifestyle risk factors and chronic diseases such as diabetes, CVDs and MetS [35–37]. Considering the high incidence of MetS among Iranian adults and the possible beneficial combined role of healthy lifestyle factors as a HLS in the prevention of some chronic diseases or deaths [38, 39] and also due to the lack of information about the relationship between HLS and risk of MetS and its components among adults, we conducted the current study to investigate the relationship between HLS and the risk of MetS and its components among apparently healthy adults living in Tabriz-Iran.

## Method

### Study population

This cross-sectional study was performed on apparently healthy adults from Tabriz, Iran. Participants were collected through announcements using the convenience method. At the beginning of the study, 400 individuals were willing to participate in the study. The relationship between dietary quality indices and obesity was regarded as a key dependent variable while calculating sample size. Using the G-power software with a correlation coefficient (r) of 0.25, = 0.05, and power of 80%, the minimal sample size was predicted to be 160. To conduct sex-stratified analysis, the final sample size of 347 (58.2% male and 41.8% female) was considered for our study [40]. Subjects participating in our study had no previous history of under or over-nutrition drug abuse, or alcohol, renal disorders, hypertension, any infections and inflammations disease, or cardiovascular disorders, thyroid diseases, and diabetes mellitus, also a trained nutritionist interviewed all study participants. In addition, participants aged < 18 or with a history of weight alteration of ≥ 5 kg in the last six months, under- or over-nutrition, or participants taking drugs that affect weight, pregnancy, lactation and participants with a special diet were excluded from the study. Tabriz University of Medical Sciences ethics committee approved this study. Before participating in the study, all patients gave their full informed satisfaction in writing. The study is reported using the STROBE guidelines. The STROBE statement is a checklist of 22 items that we consider important for good reporting of observational studies (cohort, case–control, and cross-sectional). These items relate to the article's title and abstract, introduction, methods, results, and discussion Sects. [41].

### General features and anthropometric assessment

Information about socio-economic status (SES) was obtained through a face-to-face interview of participants by the following questions: house ownership, number of family members, educational level, and occupation that were considered as separate indicator. Based on SES tertiles, collected total score was categorized in to three classes: high, middle and low [42]. Other general information such as gender, age, education level, smoking, and matrimonial status were collected by a nutritionist using a questionnaire. All surveys related to anthropometric indicators were performed in one day for each participant by an expert researcher. Body weight was assessed using a Seca scale (Germany) with a sensitivity of 0.1 kg, and height was assessed using a stadiometer with a sensitivity of 0.1 cm. A constant tension tape was used to measure waist circumference (WC) in the standing situation midway among iliac crests and lower rib margins. BMI was evaluated as weight divided by the square of height (kg/m^2^). The waist-hip ratio was assessed by dividing the WC by the hip circumference. Fat-free mass (FFM) and fat mass (FM) were evaluated by a BIA analyzer (Tanita, BC-428, Tokyo, Japan). Using a standard mercury sphygmomanometer, diastolic and systolic blood pressure (DBP and SBP) of patients were assessed twice after sitting for 15 min and their average was calculated.

### Definition of the metabolic syndrome (MetS)

In our study MetS was defined when three or more of the following components were met [43]: FBS ≥ 100 mg/dl or using anti-diabetic drugs; HDL-C < 50 mg/dl in women and < 40 mg/dl in men; TG ≥ 150 mg/dl or use of anti-lipid drugs; SBP ≥ 130 mmHg or DBP ≥ 85 mmHg or use of antihypertensive drugs and WC ≥ 95 cm for both genders, according to the new cut-off points for the Iranian adult population [44].

### Physical activity (PA) assessment

A short form of the International PA Questionnaire (IPAQ-SF) was used to assess PA levels in official Persian [45]. Translation and validation of this questionnaire were previously conducted on Iran's adult population. Based on the information provided by the individuals, the MET value was calculated by taking into account the type and number of activities performed each week as well as the time spent on them. 8.0 MET or more was defined as vigorous PA, 4.0 MET as moderate PA and walking as 3.3 MET.

### Dietary assessments

Data on dietary intake were collected using a validated semi-quantitative food frequency questionnaire (FFQ) that included 147 food items [46, 47]. Expert nutritionists asked study patients to select how many servings and frequencies of each food they used during the prior year, monthly, on a weekly, annual basis or daily basis. The portion sizes were converted to grams using a household scale. For nutrients and energy analysis, we used USDA's Food Composition Table (FCT) because the Iranian FCT does not provide much information [48].

### Healthy eating index (HEI)-2015

The HEI is a method for diet quality assessment according to dietary guidelines for Americans (DGA) recommendations [49]. HEI-2015 is composed of 13 components, which includes 4 moderation and 9 adequacy components with a total score of 100 points. The highest and lowest consumption of three adequacy components (e.g., dairy, whole grains and fatty acids) scored 10 and 0, respectively. Other six adequacy components include total fruits (fruit, fruit juice and canned fruit), whole fruits (fruits except fruit juice), total vegetables, greens and beans, total protein foods, seafood and plant proteins scored 0 and 5 in the lowest and highest intake, respectively. A maximum of 10 points was given to the lowest intake of four moderation components includes, sodium, refined grains, saturated fats and added sugars. Nevertheless, the highest intake of these components was scored as 0. Intermediate intake of every component was scored proportionally. It should be mentioned that the intermediate scores were computed proportionally. Higher total HEI-2015 scores reflecting a healthful diet and greater adherence to dietary guidelines [49, 50]. Finally, participants were divided into quintiles based on the HEI-2015 score; participants in the upper two quintiles were given a score of 1 (as a higher adherence to a healthy diet).

### Biochemical assessment

After 12 h of fasting, venous blood (10 ml) was collected from all subjects and centrifuged for 10 min at 3000 rpm, 4 °C, and kept at -80 °C until assay. Fasting blood sugar (FBS), triglyceride (TG), total cholesterol (TC), and high-density lipoprotein cholesterol (HDL-C) were assessed using a commercial kit (Pars Azmoon, Tehran, Iran). The Friedewald method was used to calculate serum low-density lipoprotein cholesterol (LDL-C) [51, 52].

### Determination of healthy lifestyle scores (HLS)

The HLS was calculated based on the method described in the previous studies [23, 31]. The score of healthy lifestyle factors for individuals was determined using four lifestyle factors, including PA (inactive/ active), diet quality (HEI-2015), smoking (no/yes), and obesity (no/yes) [31]. For computing of HLS, participants were divided into two groups based on each lifestyle factor as follows: physically low active/sedentary (< 22.5 MET/h/w) vs. physically active (≥ 22.5 MET/h/w); current smoking vs. non-current smoking; non-obese (BMI < 25 kg/m^2^) vs. overweight/obese (BMI ≥ 25 kg/m^2^). The quality of diet in individuals has been evaluated based on the AHEI-2015 diet score, which was defined in detail earlier. Healthy diets were defined as those which scored in the top 40% of HEI (upper two-fifths). A score of 1 was assigned to each of the following presenting characterizes: (BMI < 25 kg/m^2^), non-current smoking, physically active (≥ 22.5 MET/h/w), and upper two quintiles of the AHEI-2015 score. By summing up the scores that each participant received for the various components of lifestyle, we were able to construct the total HLS and the total points obtained are categorized between 0 (lowest adherence to HLS) and 4 (highest adherence to HLS).

### Statistical analyses

We used SPSS version 18.0 (Armonk, NY, IBM Corp) to analyze the data. The statistical significance of the study was determined by P-values less than 0.05. All subjects were classified based on quartiles of HLS scores. The first quartile of HLS was taken as the reference class. Percentages were used to describe qualitative variables, while mean ± standard deviation (SD) was used to describe quantitative variables. One-way analysis of variance (ANOVA) and Chi-squared tests were used to assess differences in quantitative and categorical variables across quartiles of HLS, respectively. Binary logistic regression with adjusted models was used to obtain the odds ratio (OR) and 95% confidence interval (CI) for MetS and its components across quartiles of HLS. In the first model, age and sex were adjusted. Further adjustments in the second model were made for age, sex, occupation status, educational level, marital status, BMI, and physical activity.

## Results

The average age and BMI of study participants were 40.78 ± 9.23 years old and 29.23 ± 5.97 kg/m^2^, respectively. Based on the inclusion criteria, 347 participants (58.2% male and 41.8% female) were included in this study. The incidence of MetS, high blood pressure, hypertriglyceridemia, hyperglycemia, low HDL-C, and high WC were 77.65%, 41%, 77.60%, 79.81%, 77.64%, 53.72%, and 94.61% respectively. A summary of characteristics of study participants across the quartiles of HLS are shown in Table 1. According to the results, participants in the highest quartile of HLS were older, non-smokers, and had lower WC, weight and BMI than those in the lowest quartile (*P* < 0.05). Table 2 contains the dietary intakes of participants across quartiles of HLS. The dietary intakes of fruits, vegetables, whole grains, white meat, dairy, nuts significantly increased across the quartiles of HLS with a *P* value = 0.01. No significant relation was observed between other dietary intakes across the quartiles of HLS. The data presented in Table 3 shows that individuals in the highest quartile of HLS had significantly lower FBS and TG than those in the lowest quartile (*P* < 0.05).

**Table 1:** Baseline characteristics of study population across quartiles of HLS (*n* = 470)

**Variables**		**Quartiles of HLS**			**P**
	Q1(*N* = 58)	Q2(*N* = 135)	Q3(*N* = 109)	Q4(*N* = 45)	
Age (years)	39.64 ± 8.91	39.51 ± 8.97	41.50 ± 9.49	44.28 ± 8.97	**0.01**
Gender (male)	28(48.3)	77(57)	68(62.4)	29(64.4)	0.26
BMI(kg/m^2^)	33.95 ± 3.68	31.14 ± 5.41	25.98 ± 5.21	25.27 ± 4.95	**0.01**
Weight (kg)	96.42 ± 12.86	94.34 ± 13.69	88.60 ± 15.22	88.39 ± 14.16	**0.01**
WC (cm)	109.35 ± 8.69	107.54 ± 8.97	106.12 ± 9.33	104.74 ± 10.60	**0.01**
WHR (m)	0.93 ± 0.06	0.93 ± 0.80	0.93 ± 0.80	0.94 ± 0.06	0.88
FM (kg)	35.06 ± 6.47	33.34 ± 9.32	38.08 ± 12.17	32.24 ± 9.77	0.15
FFM (kg)	61.01 ± 12.39	63.34 ± 9.32	63.04 ± 12.43	63.13 ± 10.98	0.87
Smoking (%)	16(27.6)	12(8.9)	6(5.5)	6(13.3)	**0.01**
PA (min/week)	1477.05 ± 286.83	1300.55 ± 446.64	1325.07 ± 368.56	1225.07 ± 368.56	0.05
**Marital status (%)**					0.85
Married	50(86.2)	117(86.7)	92(84.4)	37(82.2)	
**SES (%)**					0.72
Low	3(4.5)	5(3.5)	0(0)	0(0)	
Middle	33(56.8)	72(53.5)	52(47.7)	24(53.8)	
High	22(38.6)	58(43)	57(52.3)	21(46.2)	
**Education (%)**					0.18
Illiterate	0(0)	3(2.3)	0(0)	0(0)	
≤ High school/diploma	17(29.6)	31(23.2)	15(13.7)	10(23)	
≥ College degree	41(70.4)	101(74.5)	94(86.3)	35(77)	

*P*-value obtained using Chi-square test for categorical variables and one-way ANOVA for continuous variables. Continuous and Categorical variables data are presented as mean (SD) and number (percent)

*FM* Fat mass, *FFM* Fat free mass, *BMI* Body mass index, *SES* Socio-economic status, *PA* Physical activity

Bold values indicate *P* < 0.05 as the level of significance

**Table 2:** Dietary intakes of participants across Quartiles of the HLS (*n* = 470

**Variables**		**Quartiles of HLS**			**P**
	Q1(*N* = 58)	Q2(*N* = 135)	Q3(*N* = 109)	Q4(*N* = 45)	
**Macronutrients**					
Energy (kcal/d)	2263 ± 682	2252 ± 712	2174 ± 636	2382 ± 768	0.07
Carbohydrate (g/d)	461.5 ± 26.2	445.6 ± 14.1	452.8 ± 16.8	407.1 ± 22.6	0.4
Fat (g/d)	113.1 ± 7.3	102.7 ± 4.4	99.2 ± 4.4	80.1 ± 5.1	**0.01**
Protein (g/d)	99 ± 4.4	98.6 ± 3.2	92.9 ± 5	104 ± 3.8	0.38
**Food groups**					
Fruits (g/d)	383.3 ± 23.4	453.7 ± 28.1	456.7 ± 12.8	507.2 ± 14.5	**0.01**
Vegetables (g/d)	335.7 ± 7.5	365.9 ± 9.4	359.3 ± 8.6	369.4 ± 7.9	**0.01**
Dietary fiber (g/d)	67.80 ± 6.67	65.04 ± 3.07	68.23 ± 3.73	61.29 ± 4.78	0.74
Red meat (g/d)	59.8 ± 29.8	52.5 ± 22.3	33.8 ± 26.2	30.8 ± 16.1	**0.01**
Wight meat (g/d)	37.2 ± 1.5	54.1 ± 1.2	73.4 ± 1.6	92.1 ± 1.3	**0.01**
Whole grains (g/d)	132.59 ± 3.8	155.8 ± 4.2	156.6 ± 4.1	144.9 ± 3.4	0.59
Refined grains (g/d)	407.7 ± 5.8	402.78 ± 5.2	374.68 ± 4.2	343. 9 ± 4.3	0.34
Dairy (g/d)	321.2 ± 7.5	337.8 ± 9.5	322.1 ± 8.6	374.8 ± 11.1	**0.01**
Legumes (g/d)	45.61 ± 4.17	57.91 ± 5.73	64.96 ± 5.80	52.74 ± 47.10	0.21
Nuts (g/d)	53.3 ± 1.3	62.3 ± 1.6	62.1 ± 1.4	64.2 ± 1.8	**0.01**
**Nutrients**					
Sodium (mg/d)	5044.9 ± 396.2	4747.3 ± 186.9	4599.1 ± 190.4	4231.8 ± 299.5	0.31
Calcium (mg/d)	1266.8 ± 72.2	1253.3 ± 51.5	1349.2 ± 53.5	1295.5 ± 85.3	0.61
Magnesium (mg/d)	500.5 ± 24.6	544.5 ± 23.3	569.6 ± 19.4	533.0 ± 34.8	0.33
Iron (mg/d)	23.0 ± 1.31	23.5 ± 0.83	25.3 ± 1.28	21.6 ± 1.22	0.23
SFA (g/d)	30.0 ± 2.4	28.9 ± 1.2	29.5 ± 1.3	28.9 ± 2.3	0.97
PUFA (g/d)	23.0 ± 1.9	22.6 ± 1.2	23.1 ± 1.10	20.5 ± 1.9	0.74
Vitamin B1 (mg/d)	2.6 ± 0.12	2.6 ± 0.09	2.6 ± 0.09	2.4 ± 0.14	0.56
Vitamin B6 (mg/d)	2.2 ± 0.11	2.3 ± 0.08	2.4 ± 0.09	2.2 ± 0.10	0.17
Folate (µg/d)	720.8 ± 39.04	722.2 ± 27.45	739.8 ± 26.9	672.8 ± 37.76	0.51

*g/d* gram per day, Kcal/d kilocalorie per day, *HLS* Healthy life style score

Bold values indicate *P* < 0.05 as the level of significance. Values are based on mean ± standard deviation. One-way ANOVA has been used

**Table 3:** Biochemical measures across quartiles of HLS (*n* = 470)

**Variables**		**Quartiles of HLS**			**P**
	Q1(*N* = 58)	Q2(*N* = 135)	Q3(*N* = 109)	Q4(*N* = 45)	
FBS (mg/dl)	98.98 ± 31.93	91.94 ± 16.10	93.88 ± 19.37	89.75 ± 11.90	**0.02**
TC (mg/dl)	193.48 ± 40.89	192.23 ± 33.84	192.57 ± 40.32	185.84 ± 32.28	0.65
TG (mg/dl)	148.28 ± 80.61	176.11 ± 71.34	173.20 ± 66.28	129.83 ± 65.53	**0.01**
LDL-C (mg/dl)	123.64 ± 34.43	126.17 ± 32.32	123.35 ± 31.48	118.46 ± 29.23	0.5
HDL-C (mg/dl)	42.73 ± 9.10	44.14 ± 9.23	42.03 ± 9.26	44.77 ± 11.24	0.23
SBP (mmHg)	127.84 ± 11.46	122.08 ± 15.07	123.77 ± 19.21	119.87 ± 16.04	0.08
DBP (mmHg)	84.26 ± 7.82	81.11 ± 11.07	82.40 ± 13.62	80.24 ± 11.53	0.28

*Abbreviations WC* Waist circumference, *WHR* Waist hip ratio, *TG* Triglyceride, *TC* Total cholesterol, *HDL-C* High-density lipoprotein cholesterol, *FBS* Fasting blood sugar, *SBP* Systolic blood pressure, *DBP* Diastolic blood pressure

*P*-value obtained using one-way ANOVA for continuous variables, and Chi-square test for categorical variables. Continuous variables are presented as the means ± standard deviations. Bold values indicate *P* < 0.05 as the level of significance

We determined the odds of MetS and its components across the quartiles of HLS in all participants and showed main results in Table 4. In the crude model, our findings showed no substantial association of HLS with risk of MetS and its components. Albeit the probability of having MetS was lower in those who were in the fourth quartile of HLS compared to the first, however, the relationship was not significant. Our results indicate that, after modification for confounders, participants in the lowest quartile of HLS had the highest risk of MetS **(**adjusted OR: 1.24, 95% CI: 1.06–2.67, *P* = 0.03**)**. Based on the sex and age -adjusted model, the odds of high TG (OR = 0.83; 95% CI: 0.55–0.96, *P* < 0.01) and hyperglycemia (OR = 0.87; 95% CI: 0.56–0.95, *P* = 0.04) was reduced across quartiles of HLS. In addition, the results of the adjusted multivariate model presented that after adjusting age, sex, occupation status, educational level, marital status, BMI, and physical activity, the risk of high TG (adjusted OR: 0.82; 95% CI: 0.54–0.95, *P* = 0.01) and hyperglycemia **(**adjusted OR: 0.85, 95% CI: 0.55–0.96, *P* = 0.12) was decreased across the quartiles of HLS. Multivariate-adjusted ORs and 95% CIs for MetS and its components across quartiles of HLS in men and women are shown in Table 5. In men, no significant association was seen between HLS and MetS. However, after taking potential confounders into account, men in the top quartile of HLS had 14% lower odds for having high TG compared with those in the first quartile (OR: 0.86, 95% CI: 0.22–0.97). In women, there was a significant positive association between HLS and MetS after controlling for age, occupation status, educational level, marital status, BMI, energy intake and physical activity, such that women in the first quartile of HLS had 67% increased odds of MetS compared with those in the bottom quartile (OR: 1.32; 95% CI: 1.03–2.91).

**Table 4:** Odd ratio and 95% confidence interval for MetS and its components across quartiles of HLS (*n* = 470)

**Variables**		**Quartiles of HLS**			***P*-value trend**
	Q1(*N* = 58)	Q2(*N* = 135)	Q3(*N* = 109)	Q4(*N* = 45)	
**MetS**					
Crude	1 (Ref.)	1.29 (0.94–2.66)	1.19(0.81–2.82)	0.97(0.71–2.75)	0.13
Model 1	1 (Ref.)	**1.23 (1.04–2.65)**	1.17 (0.76–2.87)	0.95(0.89–2.75)	**0.01**
Model 2	1 (Ref.)	**1.24 (1.05–2.66)**	1.18(0.75–2.86)	0.96(0.86–2.77)	**0.03**
**High WC**					
Crude	1 (Ref.)	1.34 (0.57–2.44)	0.92 (0.19–2.16)	0.89 (0.69–2.34)	0.75
Model 1	1 (Ref.)	1.47 (0.54–2.35)	1.12 (0.22–2.35)	0.84 (0.89–2.45)	0.32
Model 2	1 (Ref.)	1.46 (0.53–2.36)	1.13 (0.93–2.34)	0.83 (0.88–2.44)	0.34
**High TG**					
Crude	1 (Ref.)	0.98 (0.68–1.54)	0.95 (0.69–1.32)	0.89 (0.54–1.06)	**0.02**
Model 1	1 (Ref.)	1.02 (0.72–1.48)	0.94 (0.66–1.37)	**0.83 (0.55–0.96)**	** < 0.01**
Model 2	1 (Ref.)	1.030 (0.74–1.47)	0.93 (0.65–1. 36)	**0.82 (0.54–0.95)**	** < 0.01**
**Low HDL-C**					
Crude	1 (Ref.)	1.62 (0.29–2.31)	0.67(0.28–1.58)	0.50 (0.14–1.77)	0.58
Model 1	1 (Ref.)	1.58 (0.26–2.29)	0.78(0.30–2.09)	0.60 (0.15–2.34)	0.58
Model 2	1 (Ref.)	1.56 (0.24–2.30)	0.83 (0.28–2.46)	0.56 (0.13–2.42)	0.61
**Hyperglycemia**					
Crude	1 (Ref.)	1.17(0.95–2.94)	0.98(0.59–1.35)	0.94(0.42–1.32)	0.09
Model 1	1 (Ref.)	1.19(1.00–2.54)	0.85(0.57–133)	**0.87(0.56–0.95)**	**0.04**
Model 2	1 (Ref.)	1.20(0.68–2.37)	0.90(0.58–1.38)	**0.85(0.55–0.96)**	0.12
**Elevated blood pressure**					
Crude	1 (Ref.)	0.83 (0.34–2.05)	1.10(0.41–2.92)	1.51(0.38–5.96)	0.54
Model 1	1 (Ref.)	0.76 (0.27–2.10)	1.08(0.33–3.50)	1.33(0.29–6.07)	0.62
Model 2	1 (Ref.)	1.00 (0.34–2.94)	1.76 (0.45–3.81)	2.51 (0.47–6.29)	0.2

Model 1: Adjusted for age and sex. Model 2: Model 1 + energy intake, occupation status, educational level, marital status, BMI, and physical activity

*Abbreviations*: *HDL-C* High-density lipoprotein-cholesterol, *WC* Waist circumference

*P-*values are reported based on the binary logistic regression test and are considered significant at ˂0.05. Bold Values indicate *P* < 0.05 as the level of significance

**Table 5:** Multivariate-adjusted odds ratio (95% CI) for MetS and its components across quartiles of HLS of 202 men and 145 women, participated in the cross-sectional study, Tabriz, Iran

**Variable**	**Men**				**P**	**Women**				**P**
	Q1(*N* = 58)	Q2(*N* = 135)	Q3(*N* = 109)	Q4(*N* = 45)		Q1(*N* = 58)	Q2(*N* = 135)	Q3(*N* = 109)	Q4(*N* = 45)	
**MetS**										
Crude	1 (Ref.)	1.22 (0.28–1.58)	1.29 (0.31–1.76)	1.18 (0.35–1.56)	0.12	1 (Ref.)	1.39 (0.89–2.74)	1.36 (0.85–2.27)	1.28 (0.84–2.59)	0.33
Model 1	1 (Ref.)	1.28 (0.27–1.94)	1.21 (0.34–1.98)	1.08 (0.37–1.41)	0.22	1 (Ref.)	1.35 (0.92–2.93)	1.33 (0.84–2.29)	1.29 (0.83–2.09)	0.62
Model 2	1 (Ref.)	1.29 (0.26–1.96)	1.22 (0.32–1.99)	1.09 (0.36–1.39)	0.15	1 (Ref.)	**1.32 (1.03–2.91)**	1.31 (0.83–2.78)	1.28 (0.81–2.43)	0.28
**High WC**										
Crude	1 (Ref.)	0.93 (0.56–1.45)	1.08 (0.68–1.73)	0.83 (0.56–1.35)	0.72	1 (Ref.)	1.04 (0.62–1.65)	0.82 (0.54–1.42)	0.83 (0.54–1.29)	0.68
Model 1	1 (Ref.)	0.88 (0.57–1.52)	0.98 (0.53–1.66)	0.85 (0.42–1.47)	0.9	1 (Ref.)	0.97 (0.54–1.67)	0.87 (0.49–1.45)	0.82 (0.43–1.34)	0.57
Model 2	1 (Ref.)	0.86 (0.46–1.54)	0.97 (0.55–1.64)	0.78 (0.43–1.35)	0.85	1 (Ref.)	0.96 (0.45–1.36)	0.69 (0.37–1.26)	0.68 (0.34–1.30)	0.53
**High TG**										
Crude	1 (Ref.)	1.20 (0.26–1.66)	1.23 (0.26–1.84)	0.25 (0.23–1.69)	0.26	1 (Ref.)	1.25 (0.12–1.85)	1.35 (0.16–1.24)	1.18 (0.20–3.14)	0.08
Model 1	1 (Ref.)	1.22 (0.25–1.81)	1.16 (0.23–1.77)	0.85 (0.21–1.05)	0.14	1 (Ref.)	1.28 (0.13–1.87)	1.33 (0.15–1.29)	1.19 (0.17–3.24)	0.06
Model 2	1 (Ref.)	1.24 (0.25–1.93)	1.26 (0.24–1.67)	**0.86 (0.22–0.97)**	0.03	1 (Ref.)	1.27 (0.10–1.93)	1.32 (0.12–1.37)	1.21 (0.15–3.34)	0.17
**Low HDL-C**										
Crude	1 (Ref.)	1.76 (0.25–2.29)	0.58 (0.17–1.97)	0.60 (0.10–3.49)	0.38	1 (Ref.)	1.54 (0.19–1.68)	1.02 (0.28–3.67)	0.47 (0.17–1.86)	0.68
Model 1	1 (Ref.)	1.74 (0.24–2.49)	0.68 (0.18–2.61)	0.81 (0.11–5.64)	0.72	1 (Ref.)	1.41 (0.11–1.69)	0.75(0.14–3.96)	0.22 (0.12–2.15)	0.89
Model 2	1 (Ref.)	1.63 (0.18–2.17)	0.57 (0.11–2.95)	0.53 (0.05–5.10)	0.5	1 (Ref.)	1.40 (0.10–1.67)	0.77 (0.12–4.73)	0.20 (0.11–2.24)	0.34
Hyperglycemia										
Crude	1 (Ref.)	1.20 (0.16–1.65)	1.19 (0.15–1.73)	1.13 (0.11–1.34)	0.01	1 (Ref.)	0.97 (0.27–2.37)	0.47 (0.08–2.74)	1.20 (0.28–1.19)	0.97
Model 1	1 (Ref.)	1.23 (0.16–1.84)	1.20 (0.14–1.02)	1.13 (0.12–1.01)	0.01	1 (Ref.)	0.60 (0.12–2.01)	0.38 (0.04–2.40)	1.25 (0.18–1.18)	0.97
Model 2	1 (Ref.)	1.23 (0.16–1.90)	1.20 (0.12–1.45)	1.14 (0.11–1.28)	0.02	1 (Ref.)	0.91 (0.15–2.29)	0.73 (0.06–2.57)	1.20 (0.24–1.16)	0.48
**Elevated blood pressure**										
Crude	1 (Ref.)	1.34 (0.10–1.14)	1.37 (0.09–1.45)	1.62 (0.09–4.17)	0.37	1 (Ref.)	1.15 (0.52–1.85)	1.53 (0.75–1.95)	1.83 (0.47–2.35)	0.09
Model 1	1 (Ref.)	1.27 (0.06–1.13)	1.26 (0.05–1.34)	1.37 (0.04–3.53)	0.21	1 (Ref.)	1.20 (0.44–1.98)	1.26 (0.74–1.61)	1.23 (0.63–2.52)	0.06
Model 2	1 (Ref.)	1.34 (0.07–1.50)	1.52 (0.07–3.53)	1.90 (0.06–2.12)	0.73	1 (Ref.)	1.37 (0.10–1.20)	1.57 (0.08–1.23)	1.92 (0.05–2.32)	0.43

*Abbreviations HDL-C* High-density lipoprotein-cholesterol, *WC* Waist circumference

*P-*values are reported based on the binary logistic regression test and are considered significant at ˂0.05. Bold Values indicate *P* < 0.05 as the level of significance. Model 1: Adjusted for age, energy intake. Model 2: Model 1 + occupation status, educational level, marital status, BMI, and physical activity

## Discussion

This study is of interest given that because MetS is a risk factor for diseases such as CVD, type-2 diabetes, and atherosclerosis mortality [5, 53]. The object of this cross-sectional study was to determine the relationship between HLS with MetS and its components in apparently healthy adults resident in Tabriz, Iran. We found that participants in the lowest quartile of the HLS were more likely to have MetS than those in the highest quartile. These results showed that having a higher score on the HLS compared to a lower score was associated with a reduced risk of developing MetS; however, it was not statistically considerable. There is a significant inverse association between HLS and odds of hyperglycemia, and high TG, after modification for confounding factors. Furthermore, in the current study, a lower score of HLS was associated with increased odds of MetS in women and a higher score of HLS was associated with decreased odds of high TG in men.

It is well-known that lifestyle and dietary habits have an important effect on general health [20, 22, 33, 54]. Several studies examining the relationship between lifestyle factors and risk of MetS previously focused on the role of a single factor such as physical activity, smoking, or diet; however, lifestyle factors tend to be highly interrelated, it is more valuable to have an overall approach to lifestyle as a unit variable. Our results propose that the synergistic effect of the several components of the lifestyle is probably more important than the single effect of each factor separately. Consequently, if participants are worried about their health status, the total number of healthy lifestyle factors should be enhanced. Recently, it has been investigated in some studies the combined effect of HLS on healthy aging [28], coronary disease [29], mortality [31] and MetS [34]. Nevertheless, in regard to MetS, lifestyle studies from several populations detected diverse relations. For instance, studies in North-American and Spanish subjects reported that higher adherence to healthy lifestyle habits was related to lower risks of MetS [55, 56]. Results from a cross-sectional study of 787 adults aged 45 to 75 years showed that HLS (a composite of dietary habits, smoking, PA, and alcohol consumption) was associated with a reduced risk of MetS [22]. In another study by VanWormer et al. [20] revealed that decreasing healthy lifestyle factors, mainly increased weight, alcohol consumption, and reduced vegetable/fruit intake, over two years is related to higher risk of MetS. Also, our results are further supported by recently published study that has evaluated the relationship between HLS and the risk of MetS [34]. In an Iranian cohort study a higher score of HLS was associated with a reduced risk of MetS, independent of confounding factors [34]. Notably, they determined the HLS by three ways (HLS-AHEI-2010, HLS-modified French Programme National Nutrition Santé-Guideline Score, and HLS-healthy diet pattern score), whereas we have assessed the HLS of participants in a single way (HLS-HEI-2015). However, an inverse relationship was found between a higher score of HLS and lower risk of MetS, in all three methods. It appears that the method of Patel et al. [31] may be a more appropriate method to evaluate the HLS and its association with the risk of MetS for Iranian population, since it uses the HEI to evaluate the quality of diet, which is a well-known index and has revealed an inverse association with risk of cardio-metabolic disorders in several populations [57]. However, due to differences in food habits in different countries, the HLS-modified French Programme National Nutrition Santé-Guideline Score method may not evaluate the diet quality of other populations as accurately. Another study conducted on United States showed no relationship between lifestyle factors namely alcohol drinking, PA, and smoking, and MetS [58].

This inconsistency may partially be due to varied study designs and sample size, diagnostic criteria of MetS, different methods for calculating HLS values and limited adjustment for confounding. Consequently, large-scale studies are necessary to assess the strength and presence of the relationship between the adoption of a healthy lifestyle and the risk of developing MetS. In the present study, it appears that HLS (combination of dietary habits, smoking, physical activity, and BMI) is associated with a decreasing in the risk of MetS and its components such as hyperglycemia, and high TG. Other factors such as age and sex may affect the desired effects. Moreover, participants in the highest quartile of HLS are significantly older than those in the lowest quartile. The incidence of MetS increases with age, so it may be important. In the current study, the significant positive association between low score of HLS and MetS was seen in women, but not in men.The lack of significant negative association between the fourth quartile of HLS and MetS might be due to the low number of participants in this quartile, which can, in turn, result in the wide CIs.

The underlying mechanism for this sex inconsistency is unclear; however, it might be justified by the effect of gonadal steroids on body composition and other components of MetS [59]. It is well-known that MetS is characterized by central obesity, which might be affected by gonadal steroids. A variety of cohort and cross-sectional studies also demonstrated that adherence to healthy lifestyle factors, such as a healthy diet and no alcohol, physical activity, and no smoking, can lower the risk of MetS in persons over the age of 55 and men [7, 20], but not in young adults and women [58]. According to the literature review smoking [60–62] and PA [63, 64] are related to a higher risk of developing MetS and reduction in the risk of MetS, respectively. It has been proposed that the PA has a protecting effect on decreasing the risk of MetS by the positive impact on glucose metabolism by insulin- receptor up-regulation in muscle, insulin sensitivity, and decreasing central adiposity by negative energy balance [63, 64]. Smoking is a key lifestyle risk factor contributor to insulin resistance by several mechanisms such as endothelial dysfunction, oxidative stress, hormonal imbalance, inflammation, and central adiposity [65–67]. Furthermore, smoking changes the metabolism of nutrients and lipoprotein, β-cells function, and up-regulating inflammatory factors such as C-reactive protein, which can be associated with increased risk of cardio-metabolic disorders such as dyslipidaemia, central obesity and MetS [68, 69].

The HLS was inversely associated with odds of hyperglycemia, and high TG after adjustment for potential confounders. In a study by Farhadnejad et al. [23] after adjustment for potential confounders such as energy intake, educational level, sex, age, occupational status, and marital status, the association between higher score of HLS and the lower risk of T2D was significant (OR = 0.25; 95% CI:0.10–0.61, *P* < 0.01). One of the main components of lifestyle factors affecting the risk of MetS is the food pattern of the participants. For examination of the association between HLS and risk of MetS in the study population, we have determined HLS in subjects using HLS-HEI-2015. The findings of El Bilbeisi et al. [70] in a cross-sectional study point to a higher risk of MetS, high WC, low HDL-C, high TG, and high blood pressure among patients in the lowest quartile of the HEI-2010. Low vegetable/fruit intake was also related to prevalent MetS in specific sex-ethnicity groups in the Bogalusa Heart Study [71]. Dietary pattern rich in vegetables and fruits tend to decrease inflammatory markers and are related to improvements in single subcomponents of MetS [72, 73].

It may be noted why we only considered smoking, BMI, diet quality and PA as lifestyle factors and did not consider other factors in HLS. Several previous studies on HLS have commonly used the factors we used in this study. However, some studies have included alcohol consumption in the score, but according to the culture of the studied society, alcohol consumption is prohibited in the country, and we did not collect information in this field. Considering the significant individual effect of each lifestyle factor in predicting the risk of MetS we investigated the association between the combined effect of smoking, physical activity, diet quality, and BMI and the risk of MetS in subjects who pursued a healthy lifestyle. Our findings provide relatively strong evidence that following combined healthy lifestyle factors, which are characterized by abstinence from smoking, low BMI levels, high physical activity, and a healthy dietary pattern rich in legumes and grains, vegetables and fruits, lower intake of red and processed meat and sweetened beverage, can decrease risk of MetS, hyperglycemia, and high TG. Indeed, notwithstanding the difference between previous studies and the present study regarding the definitions of HLS, the results confirmed the hypothesis that a combination of healthy lifestyle factors may have a positive effect on promoting health and reducing the risk of complex diseases such as MetS.

The main strength of this study was sex-stratified analysis. The analyses were performed by controlling for several covariates to reach an independent relationship between HLS and odds of MetS and its components. Although we adjusted for several confounding variables, residual confounding effects may have remained and actual findings may be even stronger than those acquired. Moreover, all data were collected by expert and trained interviewers with reliable and valid questionnaires. Besides, the well-known limitations of FFQ, i.e., the measurement errors and limited food choices, should be taken into account. In addition, in this study, we used HEI-2015 to assess diet quality instead of some diet components. This study has some limitations, like the cross-sectional project, that needs interpreting these results under the hypothesis that neither directionality nor causality of the relations can be recognized. Although the questionnaires were validated and interesting details were obtained from the interviews, there could still be misclassification and measurement error. One of the most important limitations of this study was the low number of participants. We did not consider stress as a lifestyle factor in the analysis, even though stress has been associated with MetS in some studies [74–76]. It is acknowledged that the results of this study may not be generalized beyond this sample of adults.

## Conclusion

The results showed that the lowest adherence to HLS was associated with higher odds MetS in women, after adjusting for all covariates. Moreover, adherence to a healthy lifestyle was related to lower odds of hyperglycemia and high TG in Iranian adults. To confirm these results, prospective studies are required.

## Data Availability

The datasets generated and/or analyzed during the current study are not publicly available due to some restrictions applied by ethics committee; but are available from the corresponding author on reasonable request.

## Abbreviations

BMI: Body mass index
CI: Confidence interval
DBP: Diastolic blood pressure
FBS: Fasting blood sugar
FFQ: Food frequency questionnaire
HDL-C: High-density lipoprotein cholesterol
IPAQ: International physical activity questionnaire
MetS: Metabolic syndrome
PA: Physical activity
SBP: Systolic blood pressure
SD: Standard Deviations
TC: Total cholesterol
TG: Triglyceride
WC: Waist circumference

## References

[1] Samson SL, Garber AJ (2014). Metabolic syndrome. Endocrinol Metab Clin.

[2] Valerio A, Nisoli E, Rossi AP, Pellegrini M, Todesco T, El Ghoch M (2020). Obesity and Higher Risk for Severe Complications of Covid-19: What to do when the two pandemics meet. J Popul Ther Clin Pharmacol.

[3] Ford ES, Giles WH, Dietz WH (2002). Prevalence of the metabolic syndrome among US adults: findings from the third National Health and Nutrition Examination Survey. JAMA.

[4] Mazloomzadeh S, Rashidi Khazaghi Z, Mousavinasab N (2018). The Prevalence of Metabolic Syndrome in Iran: A Systematic Review and Meta-analysis. Iran J Public Health.

[5] Malik S, Wong ND, Franklin SS, Kamath TV, L’Italien GJ, Pio JR (2004). Impact of the metabolic syndrome on mortality from coronary heart disease, cardiovascular disease, and all causes in United States adults. Circulation.

[6] McCracken E, Monaghan M, Sreenivasan S (2018). Pathophysiology of the metabolic syndrome. Clin Dermatol.

[7] Garralda-Del-Villar M, Carlos-Chillerón S, Diaz-Gutierrez J, Ruiz-Canela M, Gea A, Martínez-González MA (2018). Healthy lifestyle and incidence of metabolic syndrome in the SUN cohort. Nutrients.

[8] Abbasalizad Farhangi M, Vajdi M, Nikniaz L, Nikniaz Z (2020). The interaction between dietary inflammatory index and 6 P21 rs2010963 gene variants in metabolic syndrome. Eating and Weight Disorders-Studies on Anorexia, Bulimia and Obesity.

[9] Gasmi A, Noor S, Piscopo S, Menzel A (2021). Lifestyle Genetics-Based Reports in the Treatment of Obesity. Arch Razi Inst.

[10] Fardet A, Rock E (2014). Toward a new philosophy of preventive nutrition: from a reductionist to a holistic paradigm to improve nutritional recommendations. Adv Nutr.

[11] Hyder KM, Mohan J, Varma V, Sivasankaran P, Raja D. Effects of Muscle‑Specific Exercises Compared to Existing Interventions on Insulin Resistance among Prediabetes Population of South India. Journal of Natural Science, Biology and Medicine. 2021;12(2):230-.

[12] Randhawa H, Ghaedi Y, Khan S, Al-Sharbatti S. The prevalence of overweight and obesity among health care providers in the emirate of Ajman, UAE. Journal of Complementary Medicine Research. 2020;11(3):40-.

[13] Vajdi M, Nikniaz L, Pour Asl AM, Abbasalizad FM (2020). Lifestyle patterns and their nutritional, socio-demographic and psychological determinants in a community-based study: A mixed approach of latent class and factor analyses. PLoS ONE.

[14] Golubić M, Schneeberger D, Kirkpatrick K, Bar J, Bernstein A, Weems F (2018). Comprehensive Lifestyle Modification Intervention to Improve Chronic Disease Risk Factors and Quality of Life in Cancer Survivors. J Altern Complement Med.

[15] Steyn K, Damasceno A (2006). Lifestyle and related risk factors for chronic diseases. Disease and mortality in sub-Saharan Africa.

[16] Santos L. The impact of nutrition and lifestyle modification on health. European Journal of Internal Medicine. 2021. 10.1016/j.ejim.2021.09.020 34670680

[17] Park S, Kim K, Ahn HK, Kim JW, Min G, Chung BH (2020). Impact of lifestyle intervention for patients with prostate cancer. Am J Health Behav.

[18] Schroeder K, Kubik MY, Sirard JR, Lee J, Fulkerson JA (2020). Sleep is inversely associated with sedentary time among youth with obesity. Am J Health Behav.

[19] Aghajani R, Nemati N, Hojjati Zidashti Z, Bagherpour T (2020). Effect of aerobic program in the morning and afternoon on obestatin and the body composition of overweight and obese women. Journal of Chemical Health Risks.

[20] VanWormer JJ, Boucher JL, Sidebottom AC, Sillah A, Knickelbine T (2017). Lifestyle changes and prevention of metabolic syndrome in the Heart of New Ulm Project. Preventive medicine reports.

[21] Sigit FS, Trompet S, Tahapary DL, Harbuwono DS, le Cessie S, Rosendaal FR (2022). Adherence to the healthy lifestyle guideline in relation to the metabolic syndrome: Analyses from the 2013 and 2018 Indonesian national health surveys. Preventive Medicine Reports.

[22] Sotos-Prieto M, Bhupathiraju SN, Falcón LM, Gao X, Tucker KL, Mattei J (2015). A healthy lifestyle score is associated with cardiometabolic and neuroendocrine risk factors among Puerto Rican adults. J Nutr.

[23] Farhadnejad H, Teymoori F, Asghari G, Mokhtari E, Mirmiran P, Azizi F (2022). The higher adherence to a healthy lifestyle score is associated with a decreased risk of type 2 diabetes in Iranian adults. BMC Endocr Disord.

[24] Guerra ZC, Moore JR, Londoño T, Castro Y (2022). Associations of Acculturation and Gender with Obesity and Physical Activity among Latinos. Am J Health Behav.

[25] Zelenović M, Kontro T, Dumitru RC, Aksovic N, Bjelica B, Alexe DI, et al. Leisure-time physical activity and all-cause mortality: A systematic review. Revista de Psicología del Deporte. 2022;31(1).

[26] Khakpouri S, Safari M, Ghazizadeh H, Parizadeh SMR, Nematy M, Tayefi M (2019). The relationship between the healthy eating index and an alternate healthy eating index with the risk factors for cardiovascular disease in a population from northeastern Iran. Translational Metabolic Syndrome Research.

[27] Brauer P, Royall D, Rodrigues A (2021). Use of the healthy eating index in intervention studies for Cardiometabolic risk conditions: a systematic review. Adv Nutr.

[28] Atallah N, Adjibade M, Lelong H, Hercberg S, Galan P, Assmann KE (2018). How healthy lifestyle factors at midlife relate to healthy aging. Nutrients.

[29] Khera AV, Emdin CA, Drake I, Natarajan P, Bick AG, Cook NR (2016). Genetic risk, adherence to a healthy lifestyle, and coronary disease. N Engl J Med.

[30] Shan Z, Li Y, Zong G, Guo Y, Li J, Manson JE, et al. Rotating night shift work and adherence to unhealthy lifestyle in predicting risk of type 2 diabetes: results from two large US cohorts of female nurses. Bmj. 2018;363. 10.1136/bmj.k4641 PMC6247172 30464025

[31] Patel YR, Gadiraju TV, Gaziano JM, Djoussé L (2018). Adherence to healthy lifestyle factors and risk of death in men with diabetes mellitus: The Physicians' Health Study. Clin Nutr.

[32] Díaz-Gutiérrez J, Ruiz-Canela M, Gea A, Fernández-Montero A, Martínez-González MÁ (2018). Association between a healthy lifestyle score and the risk of cardiovascular disease in the SUN cohort. Revista Española de Cardiología (English Edition).

[33] Li Y, Schoufour J, Wang DD, Dhana K, Pan A, Liu X, et al. Healthy lifestyle and life expectancy free of cancer, cardiovascular disease, and type 2 diabetes: prospective cohort study. bmj. 2020;368. 10.1136/bmj.l6669 PMC7190036 31915124

[34] Mirmiran P, Farhadnejad H, Teymoori F, Parastouei K, Azizi F (2022). The higher adherence to healthy lifestyle factors is associated with a decreased risk of metabolic syndrome in Iranian adults. Nutr Bull.

[35] Farhadnejad H, Teymoori F, Asghari G, Mokhtari E, Mirmiran P, Azizi F (2022). The higher adherence to a healthy lifestyle score is associated with a decreased risk of type 2 diabetes in Iranian adults. BMC Endocr Disord.

[36] Mohammadpour S, Ghanbari M, Shahinfar H, Gholami F, Djafarian K, Shab-Bidar S (2020). The association between healthy lifestyle score with cardiorespiratory fitness and muscle strength. Int J Clin Pract.

[37] Ghanbari E, Asgari P, Seraj-Khorrami N. Effectiveness of Transcranial Direct Current Stimulation on Cravings in Overweight Individuals. International Journal of Body, Mind and Culture. 2022.

[38] Byrd D, Holmes A, Judd S, Flanders WD, Bostick RM. Associations of whole food and lifestyle-based inflammation scores with all-cause, cancer-and cardiovascular disease-specific mortality. Cancer Research. 2017;77(13_Supplement):3287-.

[39] Byrd DA, Judd S, Flanders WD, Hartman TJ, Fedirko V, Bostick RM (2020). Associations of novel dietary and lifestyle inflammation scores with incident, sporadic colorectal adenoma. Cancer Epidemiol Biomark Prev.

[40] Murray AE, McMorrow AM, O’Connor E, Kiely C, Mac Ananey O, O'Shea D (2013). Dietary quality in a sample of adults with type 2 diabetes mellitus in Ireland; a cross-sectional case control study. Nutr J.

[41] Von Elm E, Altman DG, Egger M, Pocock SJ, Gøtzsche PC, Vandenbroucke JP (2007). The Strengthening the Reporting of Observational Studies in Epidemiology (STROBE) statement: guidelines for reporting observational studies. Ann Intern Med.

[42] Khodarahmi M, Asghari-Jafarabadi M, Abbasalizad FM (2019). A structural equation modeling approach for the association of a healthy eating index with metabolic syndrome and cardio-metabolic risk factors among obese individuals. PLoS ONE.

[43] Alberti KG, Eckel RH, Grundy SM, Zimmet PZ, Cleeman JI, Donato KA (2009). Harmonizing the metabolic syndrome: a joint interim statement of the international diabetes federation task force on epidemiology and prevention; national heart, lung, and blood institute; American heart association; world heart federation; international atherosclerosis society; and international association for the study of obesity. Circulation.

[44] AZIZI F, Hadaegh F, KHALILI D, Esteghamati A, HOSSEIN PF, Delavari A, et al. Appropriate definition of metabolic syndrome among Iranian adults: report of the Iranian National Committee of Obesity. 2010. 20804311

[45] Vasheghani-Farahani A, Tahmasbi M, Asheri H, Ashraf H, Nedjat S, Kordi R (2011). The Persian, last 7-day, long form of the International Physical Activity Questionnaire: translation and validation study. Asian J Sports Med.

[46] Mirmiran P, Esfahani FH, Mehrabi Y, Hedayati M, Azizi F (2010). Reliability and relative validity of an FFQ for nutrients in the Tehran lipid and glucose study. Public Health Nutr.

[47] Esfahani FH, Asghari G, Mirmiran P, Azizi F (2010). Reproducibility and relative validity of food group intake in a food frequency questionnaire developed for the Tehran Lipid and Glucose Study. J Epidemiol.

[48] Gaeini Z, Bahadoran Z, Mirmiran P, Djazayery A (2019). The association between dietary fat pattern and the risk of type 2 diabetes. Preventive nutrition and food science.

[49] Krebs-Smith SM, Pannucci TE, Subar AF, Kirkpatrick SI, Lerman JL, Tooze JA (2018). Update of the healthy eating index: HEI-2015. J Acad Nutr Diet.

[50] Xavier J, Farias CP, Soares MSP, Silveira GdO, Spanevello RM, Yonamine M, et al. Ayahuasca prevents oxidative stress in a rat model of depression elicited by unpredictable chronic mild stress. Archives of Clinical Psychiatry (São Paulo). 2021;48:90–8.

[51] Friedewald WT, Levy RI, Fredrickson DS (1972). Estimation of the concentration of low-density lipoprotein cholesterol in plasma, without use of the preparative ultracentrifuge. Clin Chem.

[52] Rerksuppaphol L, Rerksuppaphol S. Comparison of equations for the calculation of low-density lipoprotein cholesterol in thai population. Journal of Natural Science, Biology and Medicine. 2021;12(2):224-.

[53] Tavakolizadeh J, Goli F, Ebrahimi A, Hajivosough NS, Mohseni S. Effectiveness of a Bioenergy Economy-Based Psycho-education Package on Improvement of Vegetative Function, Forgiveness, and Quality of Life of Patients with Coronary Heart Disease: A Randomized Clinical Trial. International Journal of Body, Mind and Culture. 2021:36–50.

[54] Gani IH, Al-Obaidi Z (2022). Molecular docking studies of tyrosine kinase inhibitors: Exemplified protocol to advance pharmaceutical education in medicinal chemistry. Pharm Educ.

[55] Sotos-Prieto M, Ortolá R, Ruiz-Canela M, Garcia-Esquinas E, Martínez-Gómez D, Lopez-Garcia E (2021). Association between the Mediterranean lifestyle, metabolic syndrome and mortality: a whole-country cohort in Spain. Cardiovasc Diabetol.

[56] Hershey MS, Sotos-Prieto M, Ruiz-Canela M, Christophi CA, Moffatt S, Martínez-González MÁ (2021). The Mediterranean lifestyle (MEDLIFE) index and metabolic syndrome in a non-Mediterranean working population. Clin Nutr.

[57] Chiuve SE, Fung TT, Rimm EB, Hu FB, McCullough ML, Wang M (2012). Alternative dietary indices both strongly predict risk of chronic disease. J Nutr.

[58] Bhanushali CJ, Kumar K, Wutoh AK, Karavatas S, Habib MJ, Daniel M, et al. Association between lifestyle factors and metabolic syndrome among African Americans in the United States. Journal of nutrition and metabolism. 2013;2013. 10.1155/2013/516475 PMC3572645 23431427

[59] Lovejoy J, Sainsbury A, Group SCW (2009). Sex differences in obesity and the regulation of energy homeostasis. Obes Rev.

[60] Sun K, Liu J, Ning G. Active smoking and risk of metabolic syndrome: a meta-analysis of prospective studies. 2012. 10.1371/journal.pone.0047791 PMC3474781 23082217

[61] Kim BJ, Kim BS, Sung KC, Kang JH, Lee MH, Park JR (2009). Association of smoking status, weight change, and incident metabolic syndrome in men: a 3-year follow-up study. Diabetes Care.

[62] Berlin I, Lin S, Lima JA, Bertoni AG (2012). Smoking status and metabolic syndrome in the multi-ethnic study of atherosclerosis. A cross-sectional study Tobacco induced diseases.

[63] Hidalgo-Santamaria M, Fernandez-Montero A, Martinez-Gonzalez MA, Moreno-Galarraga L, Sanchez-Villegas A, Barrio-Lopez MT (2017). Exercise intensity and incidence of metabolic syndrome: the SUN Project. Am J Prev Med.

[64] Zhang D, Liu X, Liu Y, Sun X, Wang B, Ren Y (2017). Leisure-time physical activity and incident metabolic syndrome: a systematic review and dose-response meta-analysis of cohort studies. Metabolism.

[65] van der Vaart H, Postma DS, Timens W, Ten Hacken NH (2004). Acute effects of cigarette smoke on inflammation and oxidative stress: a review. Thorax.

[66] Facchini FS, Hollenbeck CB, Jeppesen J, Chen Y-DI, Reaven G. Insulin resistance and cigarette smoking. The Lancet. 1992;339(8802):1128–30. 10.1016/0140-6736(92)90730-q 1349365

[67] Attvall S, Fowelin J, Lager I, Von Schenck H, Smith U (1993). Smoking induces insulin resistance—a potential link with the insulin resistance syndrome. J Intern Med.

[68] Stadler M, Tomann L, Storka A, Wolzt M, Peric S, Bieglmayer C (2014). Effects of smoking cessation on b-cell function, insulin sensitivity, body weight, and appetite. Eur J Endocrinol.

[69] Kolovou GD, Kolovou V, Mavrogeni S (2016). Cigarette smoking/cessation and metabolic syndrome. Clinical Lipidology.

[70] El Bilbeisi AH, El Afifi A, Djafarian K. Association of healthy eating index with metabolic syndrome and its components among type 2 diabetes patients in Gaza Strip, Palestine: A cross sectional study. Integr Food Nutr Metab. 2019;6:1e7.

[71] Yoo S, Nicklas T, Baranowski T, Zakeri IF, Yang S-J, Srinivasan SR (2004). Comparison of dietary intakes associated with metabolic syndrome risk factors in young adults: the Bogalusa Heart Study. Am J Clin Nutr.

[72] Azadbakht L, Mirmiran P, Esmaillzadeh A, Azizi T, Azizi F (2005). Beneficial effects of a Dietary Approaches to Stop Hypertension eating plan on features of the metabolic syndrome. Diabetes Care.

[73] Esposito K, Marfella R, Ciotola M, Di Palo C, Giugliano F, Giugliano G (2004). Effect of a Mediterranean-style diet on endothelial dysfunction and markers of vascular inflammation in the metabolic syndrome: a randomized trial. JAMA.

[74] Chandola T, Brunner E, Marmot M (2006). Chronic stress at work and the metabolic syndrome: prospective study. BMJ.

[75] Pyykkönen A-J, Räikkönen K, Tuomi T, Eriksson JG, Groop L, Isomaa B (2010). Stressful life events and the metabolic syndrome: the prevalence, prediction and prevention of diabetes (PPP)-Botnia Study. Diabetes Care.

[76] Kuo Wc, Bratzke LC, Oakley LD, Kuo F, Wang H, Brown RL. The association between psychological stress and metabolic syndrome: A systematic review and meta‐analysis. Obesity Reviews. 2019;20(11):1651–64. 10.1111/obr.12915 31347765

